# Supplementary material for: Isolated sign language recognition through integrating pose data and motion history images
Source: PeerJ Comput Sci. 2024 May 21;10:e2054. doi: 10.7717/peerj-cs.2054 (PMC11157617; doi:10.7717/peerj-cs.2054)
Supplement: Supplemental Information 1 — The ‘Preprocessing’ folder contains codes for tasks such as extracting pose data from video, converting this pose data into image data, and extracting motion history image features. The ‘ModelTrainEvaluation’ folder includes codes for the processes of training the deep learning model with features obtained from the Preprocessing stage, and for the combination, classification, and evaluation of these features after the training. [file peerj-cs-10-2054-s001.zip › code/Access to Datasets.docx]

**Access to Datasets**

BosphorusSign22k is a Turkish Sign Language dataset developed by researchers at Bogazici University (Camgoz et al., 2016; Özdemir et al., 2020) and is publicly accessible for academic research, provided that an End User License Agreement (EULA) is submitted by the users. Detailed contact information and the process of accessing the dataset can be found on the official dataset website: <https://ogulcanozdemir.github.io/bosphorussign22k/>.

LSA-64 (Ronchetti et al., 2016) is the Argentine Sign Language dataset provided by Universidad Nacional de La Plata and CONICET. Available at: <https://facundoq.github.io/datasets/lsa64/>.

GSL (Adaloglou et al., 2020) is the Greek Sign Language dataset maintained by the Visual Computing Lab at the Institute of Information Technology. Available at: <https://vcl.iti.gr/dataset/gsl/>.

**References**

Adaloglou, N., Chatzis, T., Papastratis, I., Stergioulas, A., Papadopoulos, G. T., Zacharopoulou, V., Xydopoulos, G. J., Atzakas, K., & Daras, P. (2020). A Comprehensive Study on Sign Language Recognition Methods. ArXiv.

Camgoz, N. C., Kindiroglu, A. A., Karabüklü, S., Kelepir, M., Sumru Ozsoy, A., & Akarun, L. (2016). BosphorusSign: A Turkish sign language recognition corpus in health and finance domains. Proceedings of the 10th International Conference on Language Resources and Evaluation, LREC 2016.

Özdemir, O., Kindiroglu, A. A., Camgöz, N. C., & Akarun, L. (2020). BosphorusSign22k Sign Language Recognition Dataset. CoRR, abs/2004.01283. https://arxiv.org/abs/2004.01283

Ronchetti, F., Quiroga, F., & Lanzarini, L. (2016). LSA64 : An Argentinian Sign Language Dataset. Congreso Argentino de Ciencias de La Computacion (CACIC).
